# Coarse-graining and the Haar wavelet transform for multiscale analysis

**DOI:** 10.1186/s42234-022-00085-z

**Published:** 2022-02-02

**Authors:** William J. Bosl, Tobias Loddenkemper, Solveig Vieluf

**Affiliations:** 1 University of San Francisco, 2130 Fulton Street, San Francisco, CA 94117 USA; 2 Department of Pediatrics, Harvard Medical School, Boston, USA; 3 Computational Health Informatics Program, Boston Children’s Hospital, Boston, USA; 4 Department of Neurology, Division of Epilepsy and Clinical Neurophysiology, Boston Children’s Hospital, Harvard Medical School, Boston, MA USA; 5 Institute of Sports Medicine, Paderborn University, Warburger Str. 100, 33098 Paderborn, Germany

**Keywords:** Haar, Wavelet, Multiscale, Coarse-graining, Multiscale entropy, Fourier transform

## Abstract

**Background:**

Multiscale entropy (MSE) has become increasingly common as a quantitative tool for analysis of physiological signals. The MSE computation involves first decomposing a signal into multiple sub-signal ‘scales’ using a coarse-graining algorithm.

**Methods:**

The coarse-graining algorithm averages adjacent values in a time series to produce a coarser scale time series. The Haar wavelet transform convolutes a time series with a scaled square wave function to produce an approximation which is equivalent to averaging points.

**Results:**

Coarse-graining is mathematically identical to the Haar wavelet transform approximations. Thus, multiscale entropy is entropy computed on sub-signals derived from approximations of the Haar wavelet transform. By describing coarse-graining algorithms properly as Haar wavelet transforms, the meaning of ‘scales’ as wavelet approximations becomes transparent. The computed value of entropy is different with different wavelet basis functions, suggesting further research is needed to determine optimal methods for computing multiscale entropy.

**Conclusion:**

Coarse-graining is mathematically identical to Haar wavelet approximations at power-of-two scales. Referring to coarse-graining as a Haar wavelet transform motivates research into the optimal approach to signal decomposition for entropy analysis.

## Background

Multiscale entropy (MSE) was first introduced as a useful quantitative property of biological signals (Costa et al., 2002), initially demonstrated as a biomarker to distinguish diseased hearts from healthy or aging hearts (Costa et al., 2005; Norris et al., 2008; Costa et al., 2008). Subsequently, MSE analysis has been used to analyze many different physiological time series to find biomarkers for many conditions, including postural control (Busa & van Emmerik, 2016), epilepsy (Aung & Wongsawat, 2021; Sathyanarayana et al., 2021), autism (Bosl et al., 2018), schizophrenia (Hasey & Kiang, 2013), Alzheimer’s Disease (Horvath et al., 2018), as well as many applications outside of physiology (Humeau-Heurtier, 2020). MSE has found increasing usefulness as a method for analyzing neurophysiological signals (Bosl et al., 2018; Gurau et al., 2017; Sathyanarayana et al., 2020). The method proposed to compute MSE involved two steps. First, the signal is decomposed into sub-signals using an averaging process called coarse-graining, then the entropy of each sub-signal is computed (Costa et al., 2002).

**Fig. 1:**
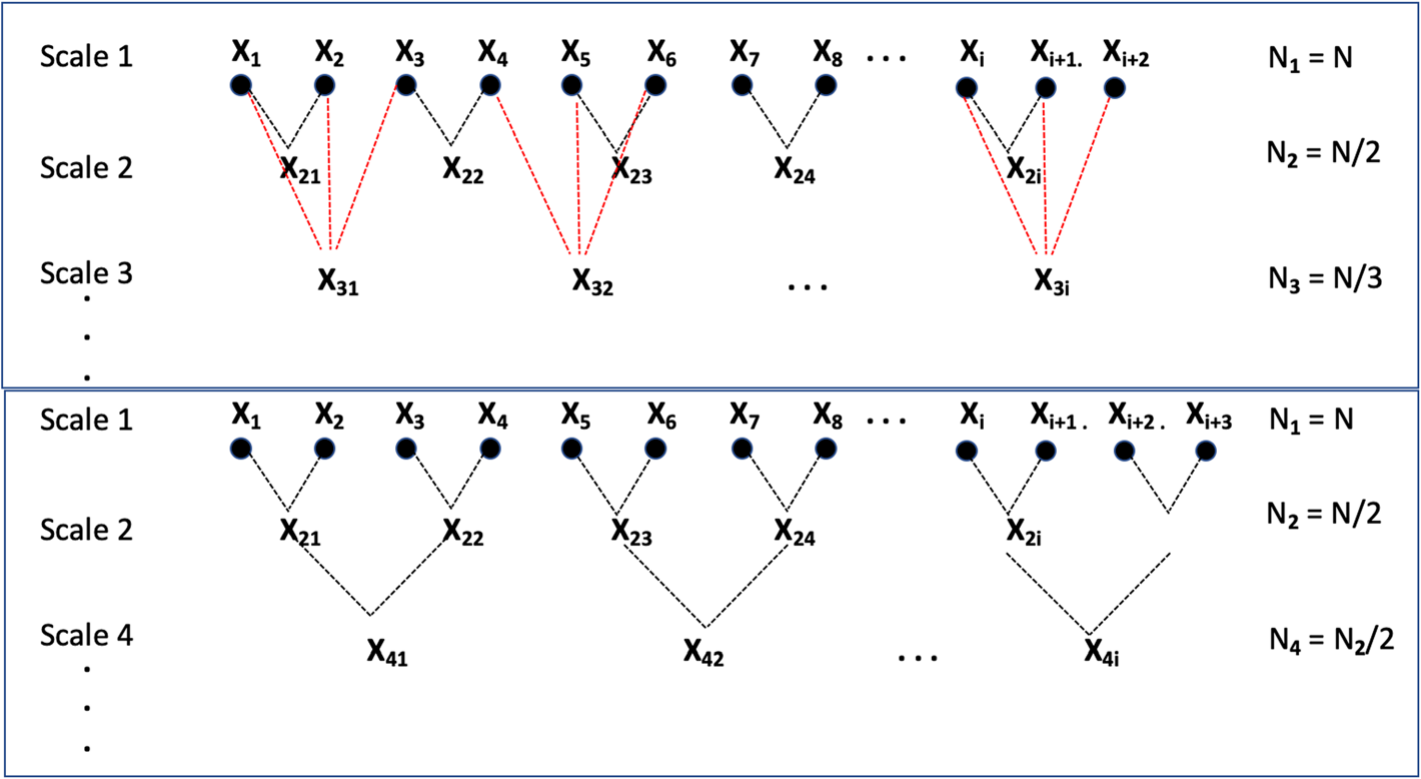
The coarse-graining procedure uses a simple averaging process to derive successive sub-signal scales (upper box). The same result can be obtained for power-of-two scales by averaging pairs of points from the preceding scale subsignal, as illustrated in the lower box

Nearly all publications involving multiscale entropy use the coarse-graining procedure as the first step in decomposing a physiological time series into smaller scales. The procedure is illustrated in Fig. 1. Historically, analysis of physiological time signals has focused on signal power in multiple, non-overlapping frequency bands or across the ‘spectrum’ of frequencies. Many methods for spectral power analysis are available, including Fourier, wavelet, and Hilbert transforms (Bruns, 2004) and more recent variations on Fourier methods such as multi-tapers that improve the time-frequency resolution of the decomposition (van Vugt et al., 2007; Babadi & Brown, 2014). If power and entropy are each considered properties of a signal or time series, the question of the relationship between the scales derived from the coarse-graining algorithm and traditional frequency bands arises.

Filtering is an almost ubiquitous step in the preprocessing of electroencephalography EEG and magnetoencephalography (MEG) data. It lies in the nature of this process itself that filtering might seriously change the appearance of the signals and thereby affect the results obtained” (Widmann et al., 2015). An ideal filter for digital signals does not exist. All filters affect signals (Cover & Thomas, 2006). Since multiscale entropy analysis first applies a filtering step, by default using coarse-graining, it is important to understand how coarse-graining affects the signal to be analyzed and, in particular, what affect it may have on the signal entropy. By demonstrating that coarse-graining is identical to the Haar wavelet transform, the well-developed mathematics of wavelet filtering can be applied to this process.

The goal of this brief report is to demonstrate that the coarse-graining procedure is mathematically identical to the ‘approximations’ that are generated by the Haar wavelet transform. This enables future research on multiscale nonlinear signal analysis to consider signal decomposition using all of the various methods commonly used for spectral power analysis in a rigorous mathematical context. Furthermore, the overlapping frequency bands derived by coarse-graining or wavelet approximations, which successively subtract higher frequencies, differ from wavelet details, which produce non-overlapping distinct frequency bands that are similar to the discrete bands used for power analysis. If entropy is computed on distinct frequency bands, then multiscale entropy could be called spectral entropy, to be compared to spectral power. Whether or not this is optimal is an open research question. Other nonlinear signal properties may also be compared with power and entropy across the same sub-signals or frequency bands.

## Methods

The coarse-graining procedure is a digital low-pass filter. Each successive scale in the coarse-graining procedure subtracts higher frequency components, leaving only the lower frequency components. The original and most common approach is to average sets of values to obtain scales: average every two points to create scale 2 subseries, average every 3 points to create scale 3 subseries, etc. (Fig. 1, upper level). A faster approach that gives only power-of-two scales is to repeatedly average every pair of points in the previous sub-signal (Fig. 1, lower level). Though not commonly done, this is obvious from simple arithmetic. We note this because power-of-two scaling is commonly used for computing wavelet levels. The power-of-two scales of coarse-graining, however computed, are mathematically *identical* to Haar wavelet transform approximations. We also note that wavelets are computed by convoluting scaled versions of the mother wavelet with the original signal. For computational efficiency, the mother wavelet is typically scaled by powers-of-two. However, this is not strictly necessary. If a Haar wavelet is scaled by successive integer factors, 2, 3, 4, and so on, and convoluted with a signal, then the resulting sub-signals are identical to the scales derived by coarse-graining. Both Haar wavelets and coarse-graining can be applied to achieve identical integer scales or power-of-two scales.

Quantitative analysis of physiological signals such as electroencephalograms (qEEG) typically involves decomposition of the signal from each sensor into non-overlapping frequency bands using some spectral decomposition method, from which the average power on each sub-signal is computed.

Many approaches, including Fourier, Wavelet, and Hilbert transforms, Empirical Mode Decomposition, multi-taper methods (used together with Fourier transforms) and others. All of these methods seek to decompose a signal into sub-signals that represent the signal at different frequency compositions or different scales. Formally, spectral analysis refers to the mathematical process of representing a function or signal as a sum of simpler functions, called basis functions, that meet certain mathematical requirements. A complete set of basis functions, called a basis set, satisfies mathematical constraints that make the set well-suited for representing other, more complex functions. For example, a set of sinusoidal functions with integer wave numbers satisfy the requirements for a basis set and are commonly used for EEG power analysis. For any set of basis functions, { *ϕ*_*n*_, *n* = 0, ±1, ±2, ±3, …}, we can write

1 $$ f(x)={\sum}_{n=-\infty}^{\infty}\left({a}_n{\phi}_n\right) $$

If the basis functions are sinusoids, then (1) represents a Fourier series for the function f(x). The coefficients, *a*_*n*_, are the amplitudes of each component function. The square of the amplitude of a sine wave is the power. Hence, the power of a specified frequency band can be conceptually found by summing the square of the coefficients of each component sine wave, although in practice more efficient algorithms are generally used.

A signal can be decomposed into multiple sub-signals, each representing a different resolution or frequency composition of the original signal, using many different mathematical approaches. For example, Fourier, Wavelet, and Hilbert transforms have been shown to be equivalent under certain conditions, though each has advantages (Bruns, 2004). A great deal of research has been devoted to finding optimal methods for spectral analysis for specific signals and goals.

Power is only one property that can be computed to characterize a signal. One of many signal properties is the sample entropy that can be computed for any time series (Humeau-Heurtier, 2020; Richman & Moorman, 2000). Just as spectral power refers to the average power in a specified frequency band, spectral entropy is an appropriate term for the entropy computed on each frequency band. The relationship between scales derived from coarse-graining and frequency bands can be made explicit.

Similar to Fourier decomposition, wavelet transforms decompose a signal using a different set of basis functions. Unlike sine waves, that are infinite, wavelet basis functions are nonzero only on a finite interval, termed ‘compact support’. The Haar wavelet was the first wavelet basis function used for signal processing. In the mid-1980s, wavelet transform analysis developed rapidly when it was found to be well-suited for seismic signal analysis (Goupillaud et al., 1984). Many different wavelet basis functions have been developed since then. An evaluation of dozens of wavelet families, each with as many as a hundred or more subtypes, found that the Daubechies 44 (db44) was most similar mother wavelet function across a variety of biological signals and potentially optimal (Rafiee et al., 2011). A comparison of wavelet bases for detecting EEG changes to multiscale entropy for a working memory task found that the sym9 wavelet gave the best results on a cross-correlation analysis (Al-Qazzaz et al., 2015). The Morlet wavelet transform is in fact identical to short-time Fourier transform (STFT) when using a Gaussian window (Bruns, 2004).

## Results and discussion

All wavelet transforms decompose a time series into pairs of subseries, each of which is half the length of the original. Mathematically this is accomplished by convolution, whereby the basis function is multiplied by the original signal while sliding along the signal. For each successive step, the basis function is scaled to be larger, thus detecting signal variation on different scales. One subseries is a running average or trend, called the ‘approximation,’ while the other subseries tracks the differences between the original points and the averages, called the ‘details.’ The approximation is a smoothed version of the signal, while the detail signal contains the finer variation that was lost in the previous approximation step. One step of a wavelet decomposition splits the signal in half by frequencies, where the approximations contain the low-frequency components and the details contain high-frequency components. An example of this process is shown in Fig. 3.

**Fig. 2:**
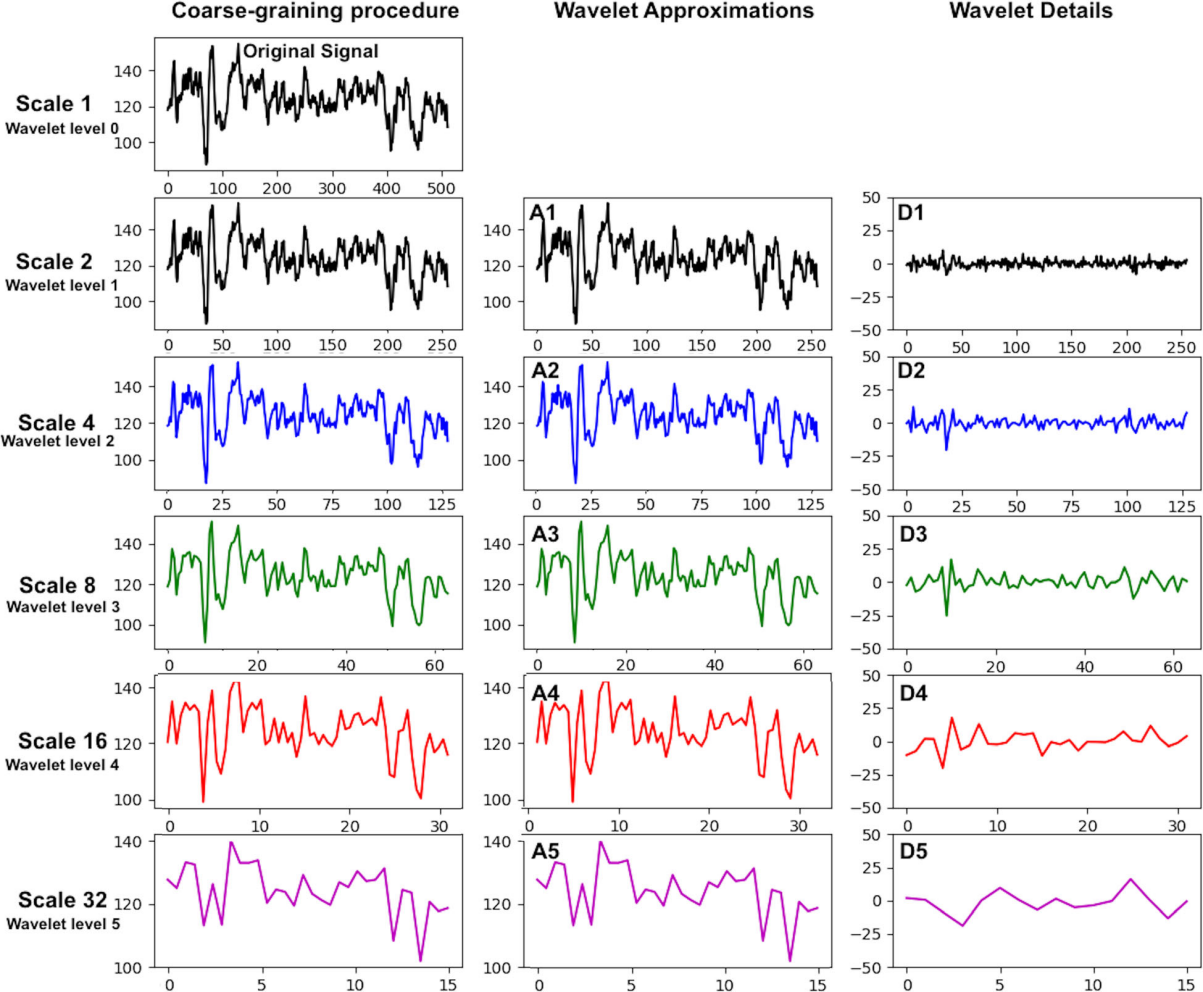
An example of a segment of an EEG time series is shown. Scales obtained by coarse-graining are shown in column 1. Haar wavelet transform approximations (column 2) and details (column 3) are also shown for equivalent levels. The horizontal axes represent points in the time series, the vertical axis is the magnitude of the field. Haar wavelet approximation levels are *identical* to power-of-two scales obtained by coarse-graining. The coarse-graining procedure does not have an equivalent to the wavelet details

The original signal in Fig. 2 is extracted from a single channel of an EEG recording and has a sampling rate of 256 Hz. It will contain frequency components up to 128 Hz, according to the Nyquist criterion, which states that a time series can only represent frequencies of up to one-half of the sampling rate. The first level of wavelet approximation contains frequency components up to 64 Hz, while the respective details contain all frequency components from 64 to 128 Hz. The process continues by successively splitting the previous approximation, producing respective approximations and details for each level.

**Fig. 3:**
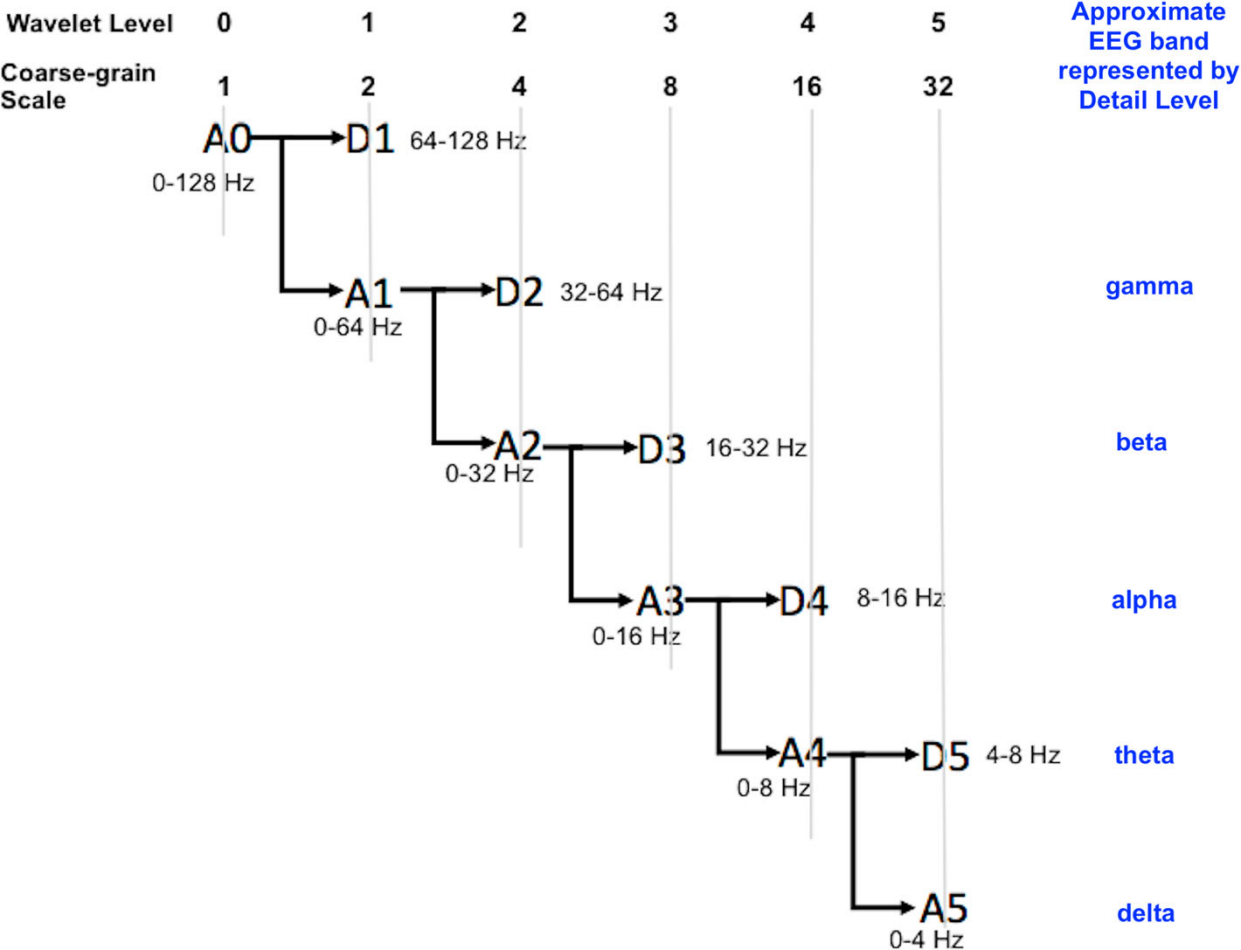
The relationship between coarse-grain scales and wavelet approximation levels is illustrated. Also shown are approximate frequency band labels associated with the wavelet detail levels Fig. 4. Sample entropy was computed using three different wavelets to decompose signals into wavelet approximations: Haar (equivalent to coarse-graining), db4, and sym9. The latter two have been cited previously as potentially optimal for analyzing biological signals. The data used for these plots were downloaded from the public PhysioNet database (www.physionet.org) (Goldberger et al., 2000). The exact files are named in each figure box. Chb files are from the CHB-MIT Scalp EEG Database, representing pediatric subjects with intractable seizures (Shoeb, 2009). The rbd file is from the CAP Sleep Database (Terzano et al., 2001). The file 0001.dat is an ECG signal from the Autonomic Aging folder in the PhysioNet Database (Schumann & Bar, 2021)

We note here that traditional power analysis computes the power of non-overlapping frequency bands. For example, electroencephalography (EEG) analysis computes the average power on five frequency bands: delta (0–4 Hz), theta (4–7 Hz), alpha (7–13 Hz), beta (13–30 Hz), and gamma (30–60 Hz). These are approximately equivalent to wavelet details (Bosl et al., 2018; Sathyanarayana et al., 2020). In contrast, coarse-graining, or Haar wavelet approximations, define overlapping bands where the higher frequencies are successively removed. Figure 3 illustrates the equivalence of coarse-grain scales and wavelet approximations, and the approximate relationship between wavelet details and traditional non-overlapping frequency bands.

The approximations produced by a Haar wavelet transform are identical to the power-of-two scales of the coarse-graining procedure. This relationship is proven mathematically by noting that the averaging filter for the Haar wavelet has a width equal to 2^n^ points on the time series, where n is the wavelet level. The coefficient for the wavelet as it slides along the length of the signal is equal to the mean of the time series values contained in its length. Each point in level A1 is found by successively averaging 2 points as the averaging filter is moved along the time series from right to left. For level 2 (or scale 4 in the coarse-graining procedure, since scale = 2^level^), the wavelet averaging filter is 2^n^ points wide, thus averaging 4 points at a time, and so on. Note that the coarse-graining scales are related to wavelet levels (A0, A1, etc) by powers of two: the scale is derived by raising 2 to the power of the wavelet level. Thus, the original signal is wavelet level 0 (A0) or scale 1 = 2^0^. Wavelet level 3 (A3) is scale 8 = 2^3^ in the coarse-graining terminology.

Suppose for any given time series there are N values. Mathematically, the values for the next level of approximation in the wavelet transform are given by:

2 $$ {a}_m=\frac{x_{2m-1}+{x}_{2m}}{2},\kern0.5em m=1\kern0.5em to\kern0.5em N/2. $$

Thus, a1 = (x_1_ + x_2_)/2, a2 = (x_3_ + x_4_)/2, a1 = (x_5_ + x_6_)/2, and so on, which is identical to the power-of-two scales in the coarse-graining procedure. The detail coefficients are given by:

3 $$ {d}_m=\frac{x_{2m-1}-{x}_{2m}}{2} $$

The reconstruction process whereby successively lower levels are reconstructed is accomplished by:

4 $$ {x}_{2m-1}=\frac{a_m+{d}_m}{2}\kern0.5em and\kern0.5em {x}_{2m}=\frac{a_m-{d}_m}{2}. $$

We note that wavelet coefficients are typically multiplied by $\sqrt{2}$ to preserve the energy or power of the signal since at each step the signal is half as many points long. Further information about wavelet algorithms can be found in wavelet texts (Addison, 2005; Walker, 2008).

**Fig. 4:**
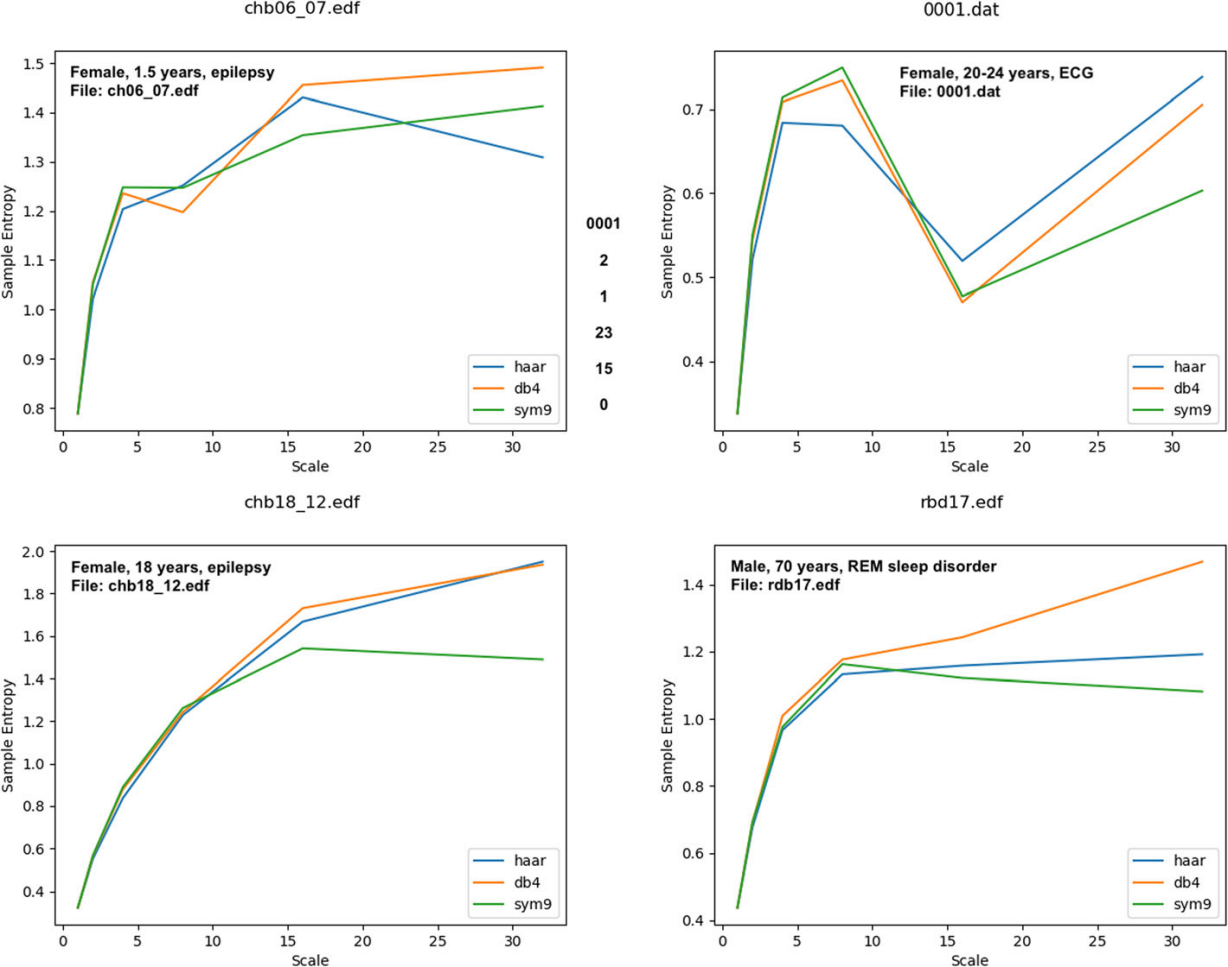
Sample entropy was computed using three different wavelets to decompose signals into wavelet approximations: Haar (equivalent to coarse-graining), db4, and sym9. The latter two have been cited previously as potentially optimal for analyzing biological signals. The data used for these plots were downloaded from the public PhysioNet database (www.physionet.org) (Goldberger et al., 2000). The exact files are named in each figure box. Chb files are from the CHB-MIT Scalp EEG Database, representing pediatric subjects with intractable seizures (Shoeb, 2009). The rbd file is from the CAP Sleep Database (Terzano et al., 2001). The file 0001.dat is an ECG signal from the Autonomic Aging folder in the PhysioNet Database (Schumann & Bar, 2021)

While power is preserved with different wavelet basis functions, the effect of different wavelet basis functions on entropy or other nonlinear measures using different wavelet basis functions has not yet been explored. In Fig. 4 multiscale entropy curves were computed for EEG and polysomnography signals using haar (equivalent to coarse-graining), db4 (Rafiee et al., 2011), and sym9 (Al-Qazzaz et al., 2015) wavelets. While the general shape of the curves is relatively the same, the values are clearly different, diverging especially in the higher scales, or lower frequency ranges. This suggests that the optimal approach to computing entropy on multiple scales remains to be fully explored.

In summary, the Haar wavelet produces subseries by averaging 2 points at a time, then 4 points at a time, then 8 points at a time, and so on. The coarse-graining procedure typically averages 2, 3, 4, 5, … points at a time. Thus, for powers of 2, the coarse-graining procedure is identical to the approximations of the Haar wavelet transform. The coarse-graining procedure does not produce any subseries equivalent to the detail subseries of a wavelet transform. The detail subseries are equivalent to the non-overlapping frequency bands produced by a typical Fourier decomposition using power-of-two multiples of the sampling rate.

## Conclusions

The relationship between scales, as used in the multiscale entropy literature, and typical frequency bands that are ubiquitous in quantitative EEG analysis is now explicit. The scales derived from coarse-graining are mathematically identical to the approximation levels derived from the Haar wavelet transform. The details produced by a wavelet transform are non-overlapping frequency bands, similar to traditional frequency bands used for EEG analysis. The Haar wavelet is only one of many wavelet basis functions that may be used for signal decomposition. A great deal of research has been done to explore optimal frequency decomposition methods for physiological signals. In future studies that use multiscale entropy, referring to the Haar wavelet transform approximation rather than ‘coarse-graining’ will place this decomposition in a rigorous context and allow comparison with alternative basis functions or with traditional spectral decomposition methods. Thus far, by default, multiscale entropy analysis has adopted the Haar wavelet decomposition. Whether or not this is optimal for multi-resolution entropy or nonlinear analysis, and why this should be different from traditional spectral decomposition used for power analysis, has yet to be explored.

## Data Availability

This paper is concerned with computational algorithms and applies generally to all time series data. Figure 2 was created using a single channel of EEG data taken from a public file randomly chosen from the PhysioNet database: https://physionet.org/content/chbmit/1.0.0/. Example data from which multiscale curves in Fig. 3 were computed were also selected from the PhysioNet database. Wavelet decompositions were computed using the example code in the Python PyWavelets package: https://pywavelets.readthedocs.io/en/latest/.

## Abbreviations

EEG: Electroencephalogram
MEG: Magnetoencephalography
MSE: Multiscale Entropy
